# Disentangling gross N_2_O production and consumption in soil

**DOI:** 10.1038/srep36517

**Published:** 2016-11-04

**Authors:** Yuan Wen, Zhe Chen, Michael Dannenmann, Andrea Carminati, Georg Willibald, Ralf Kiese, Benjamin Wolf, Edzo Veldkamp, Klaus Butterbach-Bahl, Marife D. Corre

**Affiliations:** 1 Buesgen Institute - Soil Science of Tropical and Subtropical Ecosystems, Faculty of Forest Sciences and Forest Ecology, University of Goettingen, Büsgenweg 2, 37077 Göttingen, Germany; 2 Institute for Meteorology and Climate Research, Atmospheric Environmental Research (IMK-IFU), Karlsruhe Institute of Technology (KIT), Kreuzeckbahnstrasse 19, 82467 Garmisch-Partenkirchen, Germany; 3 Department of Crop Sciences - Soil Hydrology Division, Faculty of Agricultural Sciences, University of Goettingen, Büsgenweg 2, 37077 Göttingen, Germany

## Abstract

The difficulty of measuring gross N_2_O production and consumption in soil impedes our ability to predict N_2_O dynamics across the soil-atmosphere interface. Our study aimed to disentangle these processes by comparing measurements from gas-flow soil core (GFSC) and ^15^N_2_O pool dilution (^15^N_2_OPD) methods. GFSC directly measures soil N_2_O and N_2_ fluxes, with their sum as the gross N_2_O production, whereas ^15^N_2_OPD involves addition of ^15^N_2_O into a chamber headspace and measuring its isotopic dilution over time. Measurements were conducted on intact soil cores from grassland, cropland, beech and pine forests. Across sites, gross N_2_O production and consumption measured by ^15^N_2_OPD were only 10% and 6%, respectively, of those measured by GFSC. However, ^15^N_2_OPD remains the only method that can be used under field conditions to measure atmospheric N_2_O uptake in soil. We propose to use different terminologies for the gross N_2_O fluxes that these two methods quantified. For ^15^N_2_OPD, we suggest using ‘gross N_2_O emission and uptake’, which encompass gas exchange within the ^15^N_2_O-labelled, soil air-filled pores. For GFSC, ‘gross N_2_O production and consumption’ can be used, which includes both N_2_O emitted into the soil air-filled pores and N_2_O directly consumed, forming N_2_, in soil anaerobic microsites.

N_2_O is one of the most important long-lived greenhouse gases and is expected to be the single most important ozone-depleting substance throughout the 21^st^ century^1^. Soils account, globally, for about 60% of the total N_2_O flux to the atmosphere, with 6.6 Tg N yr^−1^ from natural ecosystems and 4.1 Tg N yr^−1^ from agricultural systems^2^. Although it is generally known that microbial nitrification and denitrification in soils are the major sources of atmospheric N_2_O, it remains a struggle to disentangle and quantify gross rates of microbial N_2_O production and consumption in soil which, in turn, determine the net N_2_O flux across the soil-atmosphere interface.

Under anaerobic conditions, incomplete denitrification produces N_2_O whereas the terminal step of denitrification (i.e. the reduction of N_2_O to N_2_) consumes N_2_O. Hence, microbial N_2_O production and consumption can occur simultaneously in soil via the activities of different microorganisms or even by a single denitrifying cell^3^. In addition, within the soil profile and in the soil air-filled pores, N_2_O can be further reduced to N_2_ during its transport to the soil surface^4^,^5^,^6^. Soil physical (e.g. water or oxygen content, temperature, porosity) and biochemical factors (e.g. pH, concentrations of electron donors and acceptors) influence the balance between soil N_2_O production and consumption^7^, and consequently the net N_2_O flux to the atmosphere. Soil net N_2_O uptake has been compiled in a review^8^, which specifically refers to the net flux of N_2_O from the atmosphere to the soil and can be detected only if soil N_2_O consumption exceeds production. Soil N_2_O consumption, however, is often ignored because it is prone to be masked by the much larger N_2_O production^4^ and is difficult to measure directly (e.g. as soil N_2_ flux) against a very high (78%) atmospheric background^9^.

The static chamber method, commonly used to measure net N_2_O flux on the soil surface, cannot quantify the simultaneously occurring gross N_2_O production and consumption within the soil. One possibility to measure gross N_2_O production and consumption in soil is the ^15^N_2_O pool dilution (^15^N_2_OPD) technique, which entails adding ^15^N_2_O to the chamber headspace and subsequently measuring the changes in ^14^N_2_O and ^15^N_2_O over time^10^. So far, this ^15^N_2_OPD technique has been used in managed grassland and cropland soils and in salt marsh landscape, all located in northern California, by the same authors who first evaluated this method under field conditions^10^,^11^,^12^.

In 2013, when the first ^15^N_2_OPD measurements were reported^10^, a debate emerged as to what extent this technique is able to quantify gross N_2_O production and consumption in soil. Well & Butterbach-Bahl^13^ questioned the key assumptions of the ^15^N_2_OPD technique: the exchange and mixing of soil-derived N_2_O and ^15^N_2_O label between aerobic and anaerobic soil microsites. They argued that gross N_2_O production and consumption in soil would be underestimated if produced N_2_O was immediately reduced to N_2_ without first mixing with the ^15^N_2_O-labelled air in interconnected soil pore spaces. This may occur within denitrifier cells and between different microorganisms^3^ in anaerobic microsites, which here we infer to include not only microsites saturated with water but also isolated pores filled with or enclosed by water and water-entrapped N_2_O^14^. Yang *et al*.^15^ replied that such constraints could only occur when the soil has a high proportion of anaerobic microsites, and argued that the ^15^N_2_O label and soil-derived N_2_O are likely distributed homogeneously in the chamber headspace from which the calculation of gross N_2_O fluxes is derived. In summary, the efficacy of the ^15^N_2_OPD technique to estimate gross N_2_O production and consumption is still not settled, and so far this technique has only been compared with results from acetylene inhibition and ^15^N tracing methods. These latter methods, however, have their own limitations for determining gross N_2_O production and consumption in soil since they either modify the entire denitrification process as well as its single steps (acetylene inhibition method) or require the addition of ^15^N-labelled substrate (^15^N tracing method) with the need to label the soil homogeneously including its anaerobic microsites^9^,^16^.

To date, the enigmatic lack of measurements of gross N_2_O production and consumption in soil impedes our ability to predict N_2_O dynamics across the soil-atmosphere interface. Our study aimed to disentangle gross N_2_O production and gross N_2_O consumption in soil by comparing measurements from ^15^N_2_OPD technique and gas-flow soil core (GFSC) method. The latter is an established method that directly measures gross N_2_O production and consumption in soil by simultaneously quantifying N_2_O and N_2_ fluxes^17^ without the use of an inhibitor or ^15^N labelling of substrate^9^,^16^. We hypothesized that if the assumption of the ^15^N_2_OPD method (i.e. exchange and mixing of soil-derived N_2_O and ^15^N_2_O label between aerobic and anaerobic soil microsites) is attained, then the ^15^N_2_OPD and GFSC methods should yield comparable estimates of gross N_2_O production and consumption in soil. We tested this hypothesis using different soils from four ecosystems: grassland, cropland, beech and pine forests (Table 1), covering a range of soil biochemical characteristics as well as soil aeration status (e.g. water content and soil texture) and N availability.

## Results

**Figure 1:**
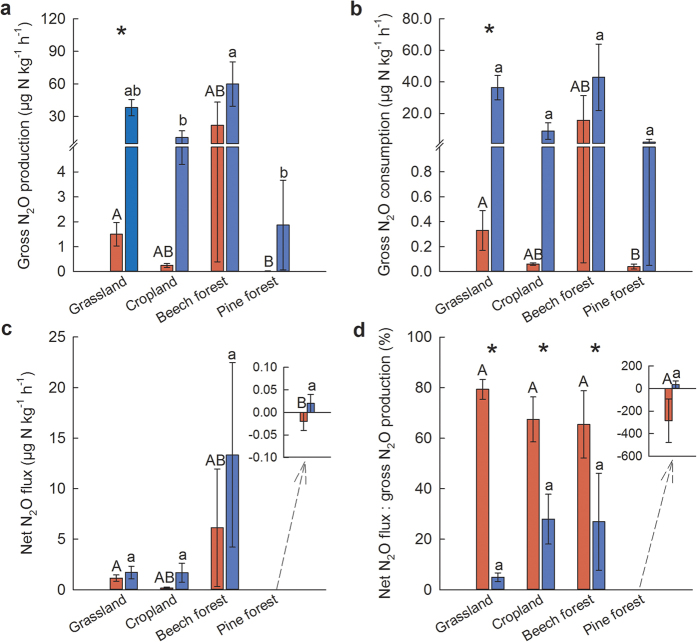
Soil gross and net N_2_O fluxes. Gross N_2_O production (**a**), gross N_2_O consumption (**b**), net N_2_O flux (**c**), and the ratio of net N_2_O flux to gross N_2_O production (**d**), measured by ^15^N_2_O pool dilution (^15^N_2_OPD; red bars) and gas-flow soil core (GFSC; blue bars). For each method, means (± s.e., n = 4 replicate sampling points) with different capital (for ^15^N_2_OPD) and small letters (for GFSC) indicate significant differences among sites (one-way ANOVA with Fisher’s LSD test at *p* ≤ 0.05 or Kruskal-Wallis ANOVA with multiple comparisons of mean ranks at *p* ≤ 0.05). For each site, asterisks above the bars indicate significant differences between the two methods (paired t test at *p* ≤ 0.05).

From the ^15^N_2_OPD measurements, gross N_2_O production and consumption rates and net N_2_O flux (Fig. 1a–c) were higher (*p* = 0.01–0.03) in the silty loam Cambisol soil in manured grassland than in the sandy Regosol soil in unmanaged pine forests, and neither differed from the sandy loam Cambisol soil in cropland or the silty loam Cambisol soil in unmanaged beech forest. For the grassland, cropland and beech forest, net N_2_O emissions accounted for 66–79% of gross N_2_O production (Fig. 1d). For the pine forest, net N_2_O uptake (Fig. 1c) was paralleled by larger gross N_2_O consumption (Fig. 1b) than gross N_2_O production (Fig. 1a); these fluxes were very small but still above our detection limit.

From the GFSC measurements, gross N_2_O production (Fig. 1a) was higher (*p* = 0.02) in the beech forest than in the cropland and pine forest and intermediate in the grassland. Gross N_2_O consumption (*p* = 0.37; Fig. 1b) and net N_2_O fluxes (*p* = 0.06; Fig. 1c) did not differ among sites. Net N_2_O fluxes accounted, on average, for only 24% of gross N_2_O production (Fig. 1d), and hence most (76%) of the produced N_2_O was further reduced to N_2_.

Although significant differences in gross N_2_O production and consumption between the ^15^N_2_OPD technique and GFSC method were only found in the grassland site (*p* = 0.02 for both; Fig. 1a,b), the fluxes measured by the GFSC method were up to two orders of magnitude larger than those measured by the ^15^N_2_OPD technique (Fig. 1a,b). The large spatial variation within each site (indicated by the large standard errors) resulted in non-statistically detectable differences between these two methods. However, for gross N_2_O production, rates measured by the ^15^N_2_OPD technique were on average 10% of those measured by the GFSC method (Fig. 1a). For gross N_2_O consumption, rates measured by the ^15^N_2_OPD technique were on average 6% of those measured by the GFSC method (Fig. 1b). Net N_2_O fluxes from the soil cores used for the ^15^N_2_OPD measurement were on average 94% of those measured by the GFSC method, which did not differ in any of the sites (*p* = 0.11–0.61; Fig. 1c). In three sites, except in the pine forest that had very low fluxes, the ratios of net N_2_O flux to gross N_2_O production measured by the ^15^N_2_OPD technique were higher (*p *< 0.01–0.05) than those measured by the GFSC method (Fig. 1d).

Soil water-filled pore space (WFPS), microbial C and N, and denitrification enzyme activity (DEA) were generally higher (*p* ≤ 0.02) in the grassland than in the pine forest (Table 2). Soil NH_4_^+^ concentrations were higher (*p *< 0.01) in the grassland and beech forest compared to the cropland, whereas soil NO_3_^−^ concentrations were higher (*p* = 0.02) in the cropland than in the grassland and pine forest (Table 2). Gross N_2_O production and consumption, measured by either the ^15^N_2_OPD technique or the GFSC method, showed positive correlations with WFPS, NH_4_^+^, microbial C and N, and DEA (R = 0.56–0.93, *p *< 0.05; Supplementary Table S1). Net N_2_O fluxes from the soil cores used for the ^15^N_2_OPD measurements correlated positively with the same soil properties (R = 0.64–0.92, *p *< 0.01; Supplementary Table S1), whereas no correlation was found with net N_2_O flux measured by the GFSC method.

## Discussion

Both the ^15^N_2_OPD and GFSC methods have been proposed to be able to measure gross N_2_O production and consumption in soils^9^,^10^. The comparable net N_2_O fluxes determined by these methods (Fig. 1c) suggest that both methods can yield similar results in terms of the net effect of concurrently occurring production and consumption of N_2_O. However, the measured gross N_2_O production and consumption rates (Fig. 1a,b), and thus the ratios of net N_2_O flux to gross N_2_O production (Fig. 1d), differed between the two methods. Hence, we reject our hypothesis that ^15^N_2_OPD technique and GFSC method yield comparable estimates of gross N_2_O fluxes.

When using the ^15^N_2_OPD technique, gross N_2_O production is determined from the dilution of ^15^N_2_O label by ^14^N_2_O produced in the soil^10^. An implicit assumption of this approach is that the headspace-labelled ^15^N_2_O that diffuses into the soil results in a homogeneous mixture of ^15^N_2_O with soil-derived N_2_O in the soil air-filled pores, which also imply that these pores must be interconnected to the soil surface for homogenous mixing to occur. Our conservative calculations of diffusive transport of ^15^N_2_O into interconnected soil air-filled pores suggest that ^15^N_2_O must have diffused into these pores and back to the headspace within 0.5 h. However, there may be two situations when gross N_2_O production and consumption will be underestimated by this method: 1) produced N_2_O is immediately consumed within denitrifier cells^3^, and 2) produced N_2_O diffuses out of denitrifier cells and is consumed by other microorganisms, which may have N_2_O reductase but cannot act on the preceding substrates of the denitrification pathway^18^, without being mixed first with the ^15^N_2_O label during the 3-hour measurement period. Both situations can occur in anaerobic microsites, which here we infer to microsites saturated with water, isolated pores filled with or enclosed by water forming a diffusion barrier, and water-entrapped N_2_O as expounded by Clough *et al*.^14^. If these situations happen, disparity between ^15^N_2_OPD and GFSC measurements would be large in a fine-textured soil with high water content whereas they would be comparable in a coarse-textured soil with low water content. The fact that our results showed the large differences between the ^15^N_2_OPD and GFSC measurements in the silty loam soil of grassland with high WFPS and they were particularly comparable in the sandy soil of pine forest with low WFPS (Fig. 1a,b; Table 2) suggest that the ^15^N_2_OPD technique was not able to quantify gross N_2_O production in these above-mentioned two situations. With the GFSC method, gross N_2_O production is measured as the sum of emitted N_2_O and N_2_, and thus those immediately consumed N_2_O to N_2_ within denitrifier cells and between different microorganims in anaerobic microsites are included in this measurement.

**Figure 2:**
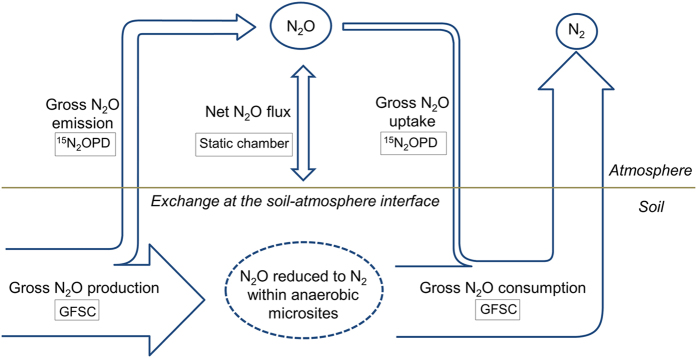
Conceptual diagram of gross N_2_O fluxes. Gross N_2_O emission and gross N_2_O uptake, measured by ^15^N_2_O pool dilution (^15^N_2_OPD), which largely includes gas exchange in interconnected air-filled pores in the soil; gross N_2_O uptake = gross N_2_O emission – net N_2_O flux. Gross N_2_O production and gross N_2_O consumption, measured by gas-flow soil core (GFSC), which encompasses the soil air-filled pores as well as anaerobic microsites (e.g. soil micro spots saturated with water, isolated pores filled with or enclosed by water, and water-entrapped N_2_O); gross N_2_O consumption = N_2_ emission, and gross N_2_O production = gross N_2_O consumption + net N_2_O flux.

We summarize our results into a conceptual model in order to illustrate two decoupled pathways of N_2_O production and consumption in soil (Fig. 2). In the first pathway, N_2_O is produced in anaerobic microsites and reduced immediately to N_2_ without first mixing with the ^15^N_2_O label. Based on our results, only the GFSC method but not the ^15^N_2_OPD technique was able to quantify this pathway. The second pathway covers the soil-derived N_2_O that diffuses into the interconnected soil air-filled pores and mixes with the ^15^N_2_O label, which was captured by the ^15^N_2_OPD technique. Even if the N_2_O that has moved into the soil air-filled pores is being consumed during its diffusion towards the soil-atmosphere interface^4^, as long as the produced N_2_O mixes with the ^15^N_2_O label this can be included in the ^15^N_2_OPD calculations of gross N_2_O production. It is clear that both ^15^N_2_OPD and GFSC methods yield complementary important information, and thus a differentiation in the use of terminologies is needed. Since the ^15^N_2_OPD technique reflects the N_2_O dynamics in the gas phase of the soils and its exchange with the atmosphere, we propose to use the terms ‘gross N_2_O emission’ and ‘gross N_2_O uptake’ to denote the gross N_2_O fluxes in interconnected soil air-filled pores measured by this method. Since the GFSC method measures gross N_2_O fluxes not only in interconnected soil air-filled pores but also in anaerobic microsites, we propose that the terms ‘gross N_2_O production’ and ‘gross N_2_O consumption’ be used (Fig. 2). Below we will use these proposed terminologies to distinguish between the processes measured by these two methods.

It is important to point out that the ^15^N_2_OPD technique is able to yield information on gross N_2_O uptake from the atmosphere to the soil. For years there has been a discussion on the importance of N_2_O uptake in the soil from the atmosphere and substantial progress has been hampered because until now only the net N_2_O fluxes on the soil surface can be routinely measured with inexpensive static chamber method. With the ^15^N_2_OPD technique, we now have an operational approach that can be used for field measurements and can separate the net N_2_O fluxes across the soil-atmosphere interface into gross N_2_O emission and gross N_2_O uptake. It is a significant advancement since this technique will allow us to investigate the factors that control N_2_O uptake by soils under actual field conditions, which is a commonly unquantified sink of ecosystem N budgets.

Moreover, our results contrast to the notion that substantial N_2_O uptake only happens in soils with net negative N_2_O flux. This was shown by the larger gross N_2_O uptake (measured by ^15^N_2_OPD technique) in the grassland that had larger net N_2_O emissions than in the pine forest that had a net negative N_2_O flux (Fig. 1b,c). The positive correlations of gross N_2_O uptake with soil biochemical characteristics (Supplementary Table S1) suggest that high gross N_2_O uptake occurs in soils with high microbial activity and high substrate availability (Table 2). The ratios of net to gross N_2_O emissions (66–79% in grassland, cropland and beech forest; Fig. 1d) were similar to the values reported by Yang *et al*.^10^ and Yang and Silver^12^ from managed grassland and cropland in California (net to gross N_2_O emission ratio of 68–70%). These generally comparable ratios may open the possibility of making estimates of gross N_2_O emissions and uptake based on measured net N_2_O emissions.

The large fraction of gross N_2_O production that was consumed to N_2_ (measured by GFSC method) suggests that gross N_2_O production and consumption were closely coupled, which is in line with our aforementioned deduction (i.e. most N_2_O was immediately reduced to N_2_ in anaerobic microsites). Hence, the similar correlations found for gross N_2_O production and consumption with soil biochemical characteristics (Supplementary Table S1) as those found for gross N_2_O emission and uptake (measured by ^15^N_2_OPD technique) suggests that these gross N_2_O fluxes were regulated by the same process, denitrification^4^.

Our findings show that whereas the ^15^N_2_OPD technique is a valuable tool to separate net N_2_O flux across the soil-atmosphere interface into gross N_2_O emission and uptake, it did not allow measuring a large part of gross N_2_O production and consumption in anaerobic microsites. In order to avoid misinterpretations of terminologies, we propose that the terms ‘gross N_2_O emission and uptake’ should be used for gross N_2_O fluxes measured with the ^15^N_2_OPD technique and ‘gross N_2_O production and consumption’ should be used for gross N_2_O fluxes measured with the GFSC method.

## Methods

### Study sites and soil sampling

Soil samples were collected from four ecosystems: grassland, cropland, beech and pine forests, covering different vegetation, soil types and climatic conditions (Table 1). The montane grassland is manured 2–3 times a year and cut for hay three times a year^19^. The cropland is a conventional corn-winter wheat rotation. The unmanaged beech forest (*Fagus sylvatica)* is 163 years old^20^, and the unmanaged Mediterranean pine forest (*Pinus pinaster*) is 52 years old^21^.

At each site, we selected four sampling points as replicates with a minimum distance of 25 m from each other. At each replicate, eight intact soil cores (250 cm^3^ each) were taken using stainless-steel cores (8 cm diameter, 5 cm height): four of which were used for the ^15^N_2_OPD measurement and the other four for the GFSC measurement. The ^15^N_2_OPD measurement was conducted concurrently with the GFSC measurement, such that the soil cores for these two methods were handled similarly in all aspects. Neither soil moisture nor substrate level was adjusted.

### ^15^N_2_O pool dilution

Four intact soil cores were placed in an incubation glass (6.6 L volume), equipped with Luer-lock stopcock for gas sampling. Upon closure of the incubation vessel, we injected into the chamber headspace 7 mL of ^15^N_2_O label gas, containing 100 ppmv of 98% single labelled ^15^N-N_2_O, 275 ppbv sulfurhexafluoride (SF_6_, as a tracer for physical loss of N_2_O) and the rest as synthetic air. This injected amount increased the N_2_O concentration in the headspace by approx. 106 ppbv N_2_O with 12.5 atom% ^15^N enrichment and SF_6_ concentration of 292 pptv. At 0.5, 1, 2, and 3 h following label gas injection, 100 mL and 12 mL gas samples were taken out and stored in pre-evacuated 100 mL glass bottles and 12 mL glass tubes (Exetainer; Labco Limited, Lampeter, UK), respectively, with rubber septa. The sampled air volume was then replaced with 112 mL of a gas mixture (80% helium and 20% oxygen) to maintain the headspace at atmospheric pressure and oxygen concentration, without altering the isotopic composition of the headspace N_2_O. The dilution that this replacement caused was accounted for in the calculations. The 100 mL gas samples were used to analyze isotopic composition using an isotope ratio mass spectrometer (IRMS) (Finnigan Delta^plus^ XP, Thermo Electron Corporation, Bremen, Germany). The 12 mL gas samples were used to measure N_2_O and SF_6_ concentrations using a gas chromatograph equipped with an electron capture detector (GC 6000 Vega Series 2, Carlo Erba Instruments, Milan, Italy). The detection limit of the entire measurement set-up and instrument precision was <0.9 ppbv N_2_O h^−1^.

We modeled the vertical diffusive transport of ^15^N_2_O label through the 5 cm long soil cores, using the diffusion equation 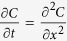 in which C, t and x denote concentration, time and path length, respectively^22^. The free-air N_2_O diffusion coefficient at 15 °C, 0.1582 cm s^−1^, was used and adjusted for soil tortuosity based on the air-filled porosity^23^, which was calculated using the measured bulk density and gravimetric moisture contents. Our most conservative calculations, using the lowest air-filled porosity and assuming an impervious boundary condition at bottom of the soil cores, showed that the ^15^N_2_O label had diffused into the 5 cm long soil cores and back to the headspace within 0.5 h. Thus, our sampling interval during the 3-hour measurement period was sufficient to allow mixing of the label gas with the soil-derived N_2_O in interconnected air-filled pores and to quantify the changes in N_2_O concentrations and ^15^N_2_O enrichments in the headspace.

Gross N_2_O emission rate was calculated using the following equations modified from Yang *et al*.^10^:


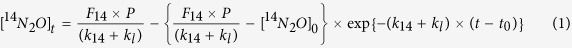



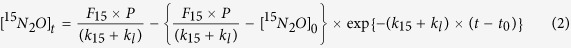


where [^14^N_2_O]_t_ is the concentration of ^14^N_2_O at time t, calculated as the product of N_2_O concentration and ^14^N-N_2_O atom%; [^15^N_2_O]_t_ is the concentration of ^15^N_2_O, calculated as the product of N_2_O concentration and ^15^N-N_2_O atom% excess, assuming that the ^15^N isotopic composition of background N_2_O is 0.3688 atom%^10^; t represents the time of gas sampling from the headspace; F_14_ represents the ^14^N_2_O mole fraction (0.997) and F_15_ represents the ^15^N_2_O mole fraction (0.003) of emitted N_2_O; *k*_14_ and *k*_15_ represent the first-order rate constants of ^14^N_2_O and ^15^N_2_O reduction to N_2_, respectively, calculated based on the fractionation factor (*α* = *k*_15_/*k*_14_) that has an average value of 0.9924 ± 0.0036 in literature^10^; *k*_*l*_ represents the first-order rate constant for loss of inert transport tracer, SF_6_; P is gross N_2_O emission rate. The *k*_14_ and *k*_15_ represent the biological loss, and *k*_l_ represents the physical loss. Since the changes of their concentrations in the headspace are simultaneously affected by biological consumption and physical loss, we used the sum of these constants (*k*_14_ + *k*_*l*_ or *k*_15_ + *k*_*l*_) in the above equations.

We estimated the parameters for P and *k*_15_ by simultaneously fitting the measured [^14^N_2_O]_t_ and [^15^N_2_O]_t_ with equation (1) and (2). The best fit of [^14^N_2_O]_t_ and [^15^N_2_O]_t_ was found using the least square approach and minimizing the following error function:


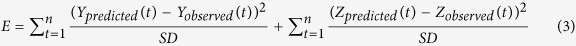


where E is minimal weighted error (E); Y, Z and n indicate ^14^N_2_O, ^15^N_2_O concentrations, and the number of measurements, respectively; SD refers to the standard deviation of the observed concentrations over the course of measurements^24^,^25^. Equation (3) was minimized using the ‘fminsearchbnd’ function in MATLAB (MathWorks, Version R2011b, USA). Gross N_2_O uptake was calculated as the difference between gross N_2_O emission and net N_2_O flux^10^.

### Gas-flow soil core

The GFSC method is a fully automated, direct and sensitive quantification of the change of N_2_O and N_2_ concentrations in the headspace above the soil cores. The soil air of the four soil cores and the headspace of the incubation vessel were completely replaced by a gas mixture consisting of 20% O_2_ (purity grade of 5.5), 80% He (purity grade of 5.0), N_2_O (400 ppbv) and N_2_ (25 ppmv). This complete exchange was done by automated repeated cycles of evacuation and gas purging, achieved through a built-in purging system in an extremely air-tight chamber that is connected directly to a gas chromatograph (Shimadzu GC-17A, Shimadzu, Munich, Germany)^17^,^26^,^27^,^28^. Eighteen hours of evacuation-purging cycles ensure a complete removal of the background atmospheric air^27^, after which the headspace and tubing connections to the gas chromatograph were further purged for three hours. Subsequently, the system switched to a static chamber mode, and the headspace air of the incubation vessel was analyzed hourly over four hours through a directly connected gas chromatograph with an electron capture detector for N_2_O analysis and a pulse discharge He ionization detector (Vici AG, Schenkon, Switzerland) for N_2_ analysis^26^. To sample the headspace, a slight overpressure was created by injecting 40 mL of the He-based gas mixture to the headspace, directing headspace air to the sampling loops^26^. The dilution of this non-intrusive overpressure sampling technique was accounted for in the calculation of N_2_O and N_2_ concentrations^26^. In order to achieve the best possible tightness of the incubation system against intrusion of atmospheric N_2_, all tubing connections, valves as well as the entire incubation vessel were placed under water. Before starting the N_2_O and N_2_ measurements, the air-tightness of the system was always checked with an empty incubation vessel, which was connected in parallel with the measuring vessel. Based on the sensitivity and repeatability of the gas chromatograph measurements, the detection limits were <0.03 ppmv h^−1^ for N_2_ and <0.45 ppbv h^−1^ for N_2_O. The measured N_2_ flux from the soil equals to gross N_2_O consumption whereas the sum of N_2_ and N_2_O fluxes equals to gross N_2_O production^17^,^26^,^27^,^28^.

### Soil controlling factors

Soil water content (one-day oven-drying at 105 °C and expressed as WFPS using 2.65 g cm^−3^ as particle density and the measured bulk density; Table 1), NH_4_^+^ and NO_3_^−^ concentrations (0.5 M K_2_SO_4_ extraction), and microbial biomass C and N (CHCl_3_ fumigation-extraction) were determined from the soil cores immediately after the gas measurements. NH_4_^+^ and NO_3_^−^ concentrations in the soil extract were determined using continuous flow autoanalyzer (Skalar Scan plus system, Skalar Analytical B.V., Breda, Netherlands). Microbial biomass C and N were determined as the difference in 0.5 M K_2_SO_4_-extractable organic C and N (analyzed using persulfate oxidation with an infrared detector; Multi N/C 3100 TOC/TNb-Analysator, Analytik Jena, Jena, Germany) between the fumigated and unfumigated soils divided by k_EC_ = 0.45 and k_EN_ = 0.68^29^. DEA was determined from the N_2_O produced during an anaerobic incubation with glucose and NO_3_^−^ added in excess and acetylene inhibited N_2_O reduction of to N_2_^30^.

### Statistical analysis

The above soil properties, determined separately from the soil cores used for ^15^N_2_OPD and GFSC measurements, did not differ between these two measurements (*p* > 0.05; paired t test); thus, the values from the two measurements were averaged to represent a replicate sampling point. Data sets were first tested for normal distribution (Shapiro-Wilk’s test) and equality of variance (Levene’s test). We used log-transformation for variables with non-normal distributions or unequal variances and assessed the differences in gross N_2_O fluxes and soil properties among sites using one-way analysis of variance (ANOVA) with Fisher’s least significant difference test. When none of the data transformations were able to attain normal distribution and equality of variance, differences among sites were tested using the Kruskal-Wallis ANOVA with multiple comparisons test. The differences in gross and net N_2_O fluxes between the ^15^N_2_OPD and GFSC methods for each site were assessed using the paired t test. Relationships of gross N_2_O fluxes with soil properties were assessed using spearman rank correlation test. Statistical significance was set at *p* ≤ 0.05. Statistical analyses were conducted using SPSS (SPSS, Chicago, Illinois, USA).

## Additional Information

**How to cite this article**: Wen, Y. *et al*. Disentangling gross N_2_O production and consumption in soil. *Sci. Rep.* **6**, 36517; doi: 10.1038/srep36517 (2016).

**Publisher’s note:** Springer Nature remains neutral with regard to jurisdictional claims in published maps and institutional affiliations.

## Supplementary Material

Supplementary Information

## Figures and Tables

**Table 1:** Site characteristics.

Site characteristics	Grassland	Cropland	Beech forest	Pine forest
Location	47.57°N, 11.03°E	48.19°N, 11.96°E	51.76°N, 9.58°E	43.72°N, 10.28°E
Mean annual temperature (°C)	6.7	8.5	7.3	14.1
Mean annual precipitation (mm)	1373	1029	1100	918
Elevation (m above sea level)	870	510	510	10
Vegetation/Crop	*Poaceae*; *Taraxacum*	*Zea mays*	*Fagus sylvatica*	*Pinus pinaster*
Soil type	Haplic Cambisol	Calcaric Cambisol	Dystric Cambisol	Calcareous Regosol
Soil texture (% sand/silt/clay)	10/68/23	30/52/18	12 / 54/34	93/3/4
Soil bulk density (g cm^−3^)	0.59	1.17	0.64	1.30
Soil pH	7.1	6.7	3.8	5.7
Soil total organic carbon (g C kg^−1^)	135	20	127	10
Soil total nitrogen (g N kg^−1^)	8.0	1.7	6.6	0.7
Soil C:N ratio	16.9	11.8	18.9	13.5

Soil characteristics in the grassland, cropland and pine forest sites were measured in the top 10 cm of mineral soil^19,21^; in the beech forest site, these were measured in the top 5 cm of mineral soil.

**Table 2:** Soil physical and biochemical characteristics in the top 5 cm, determined from the soil cores immediately after the measurement of gross N_2_O fluxes.

Soil characteristics	Grassland	Cropland	Beech forest	Pine forest
Water-filled pore space (%)	79 ± 1 a	57 ± 2 ab	70 ± 14 ab	25 ± 1 b
NH_4_^+^ (mg N kg^−1^)	4.34 ± 0.97 a	0.66 ± 0.12 b	2.35 ± 0.37 a	1.30 ± 0.18 ab
NO_3_^−^ (mg N kg^−1^)	1.00 ± 0.14 b	5.42 ± 0.60 a	4.17 ± 2.14 ab	0.71 ± 0.38 b
Microbial C (g C kg^−1^)	3.26 ± 0.13 a	0.76 ± 0.03 c	2.68 ± 0.24 ab	1.72 ± 0.10 bc
Microbial N (mg N kg^−1^)	211.02 ± 4.84 a	69.22 ± 0.90 c	160.90 ± 11.35 ab	98.70 ± 5.37 bc
Denitrification enzyme activity (g N kg^−1^ h^−1^)	5.16 ± 0.64 a	0.21 ± 0.07 bc	0.83 ± 0.17 ab	0.00 ± 0.00 c

Means ± s.e. (n = 4) within each row followed by different letter indicate significant differences among sites (one-way ANOVA with Fisher’s LSD test at *p* ≤ 0.05 or Kruskal-Wallis ANOVA with multiple comparisons of mean ranks at *p* ≤ 0.05.
