# Supplementary material for: Disentangling gross N2O production and consumption in soil
Source: Sci Rep. 2016 Nov 4;6:36517. doi: 10.1038/srep36517 (PMC5109911; doi:10.1038/srep36517)
Supplement: Supplementary Information [file srep36517-s1.pdf]

Disentangling gross N_2O production and consumption in soil

Yuan Wen[1†], Zhe Chen[2†], Michael Dannenmann[2], Andrea Carminati[3], Georg Willibald[2], Ralf Kiese[2], Benjamin Wolf[2], Edzo Veldkamp[1], Klaus Butterbach-Bahl[2] and Marife D. Corre[1*]

[1]Buesgen Institute - Soil Science of Tropical and Subtropical Ecosystems, Faculty of Forest Sciences and Forest Ecology, University of Goettingen, Büsgenweg 2, 37077 Göttingen, Germany

[2]Institute for Meteorology and Climate Research, Atmospheric Environmental Research (IMK-IFU), Karlsruhe Institute of Technology (KIT), Kreuzeckbahnstrasse 19, 82467 Garmisch-Partenkirchen, Germany

[3]Department of Crop Sciences - Soil Hydrology Division, Faculty of Agricultural Sciences, University of Goettingen, Büsgenweg 2, 37077 Göttingen, Germany

[*]Corresponding author: mcorre@gwdg.de

†These authors contributed equally to this work.

Supplementary Table S1. Relationships between soil physical and biochemical characteristics and gross N_2O production and consumption, measured by $^{15}N_2O$ pool dilution technique and gas-flow soil core method.

**Explanatory soil variables**	**$^{15}N_2O$ pool dilution**			**Gas-flow soil core**		
	Gross N_2O production	Gross N_2O consumption	Net N_2O flux	Gross N_2O production	Gross N_2O consumption	Net N_2O flux
pH	-0.09	-0.08	-0.07	-0.19	-0.31	-0.17
Water-filled pore space	0.92**	0.85**	0.92**	0.58*	0.56*	0.32
NH_4^+-N	0.69**	0.68**	0.64**	0.70**	0.65*	0.34
NO_3^--N	0.20	0.10	0.22	-0.17	-0.17	0.17
Microbial C	0.77**	0.72**	0.77**	0.60*	0.60*	0.16
Microbial N	0.74**	0.73**	0.71**	0.66**	0.69**	0.27
Denitrification enzyme activity	0.93**	0.80**	0.91**	0.83**	0.74**	0.42

Correlations were assessed using Spearman rank correlation test; n = 16; * indicates $p \leq 0.05$, and ** indicates $p \leq 0.01$.
